# Protein kinase C Inhibitors selectively modulate dynamics of cell adhesion molecules and cell death in human colon cancer cells

**DOI:** 10.1080/19336918.2018.1530933

**Published:** 2018-10-11

**Authors:** Muzaffer Dükel, Zehra Tavsan, Duygu Erdogan, Deniz Erkan Gök, Hulya Ayar Kayali

**Affiliations:** a Moleculer Biology and Genetic Department, Faculty of Art and Science, Mehmet Akif Ersoy University, Burdur, Turkey; b Izmir Biomedicine and Genome Center, Izmir, Turkey; c Izmir International Biomedicine and Genome Institute, Dokuz Eylül University, Izmir, Turkey; d Biochemistry Division, Chemistry Department, Science Faculty, Dokuz Eylul University, Izmir, Turkey

**Keywords:** Colon cancer, protein kinase C, cell adhesion molecules, reactive oxygen species, cell death

## Abstract

During development of colon cancer, Protein Kinase Cs (PKCs) are involved in regulation of many genes controlling several cellular mechanisms. Here, we examined the changes in cell adhesion molecules and PKCs for colorectal cancer progression. We identified that PKCs affected expression of EpCAM, claudins, tetraspanins. Treatment with low concentrations of PKC inhibitors resulted in decreased cell viability. In addition, immunoblotting and qRT-PCR analysis showed that apoptosis was inhibited while autophagy was induced by PKC inhibition in colon cancer cells. Furthermore, we observed decreased levels of intracellular Reactive Oxygen Species (ROS), lipid peroxidation and protein carbonyl, confirming the ROS-induced apoptosis. Taken together, our results reveal that PKC signalling modulates not only cell adhesion dynamics but also cell death-related mechanisms.

**Abbreviations:** PKC: Protein Kinase C; EpCAM: Epithelial cell adhesion molecule; FBS: fetal bovine serum; MTT: 3-(4,5-dimethylthiazol-2-yl)-2,5-diphenyltetrazolium bromide); CAM: cell adhesion molecule; ROS: reactive oxygen species

## Introduction

Colon cancer is the second most common cancer in women and the third most common in men. The incidence rates are higher in more developed countries than in less developed ones. However, the mortality rate is higher in the latter, which indicates poor survival [1]. Cancer development is a multi-stage and multi-factorial process involving a series of events affecting proto-oncogenes or tumour-suppressor genes, and generally occurs over an extended period [2]. During this process, Protein Kinase Cs (PKCs) act as central players in signal transduction. Members of the PKC family contribute to the regulation of various cellular processes including apoptosis, cell survival, proliferation and transformation [3,4]. Increasing evidence supports that the change in the expression of PKCs may play an important role in colon cancer development, and the deficiency in PKCs activity is associated with altered cell proliferation, survival and anti-apoptotic pathways in colon cancer and other cancer types [5]. Therefore, PKCs have become the subject of many cancer studies. Several drugs targeting PKC isoforms have been synthesized to modulate PKC activity and many clinical trials have been conducted [6]. Also, the maintenance, promotion or disruption of cell adhesion are particularly important processes in colon cancer progression and metastasis. Among the cell adhesion molecules, epithelial cell adhesion molecule (EpCAM) is a pleitropic molecule that is highly expressed in a variety of carcinomas, especially in colon cancer [7], and is an immunotherapeutic target for several malignancies [8]. Claudins, one of the major transmembrane proteins that contribute to formation of tight junctions, have potential role in the regulation of tumorigenesis [9,10]. Mess et al. observed that claudin-1, claudin-3, and claudin-4 are overexpressed in colorectal cancer [11]. In addition, in other studies, EpCAM was identified to be important for modulating the function of tight junctions, which is vital for metastasis of cancer cells via co-localization of selected claudins [12]. Besides, there are more than 30 mammalian members of the tetraspanins family proteins which are expressed on the cell surface or within intracellular organelles and granules. Each family member has a distinct pattern of expression depending on the cancer type [13,14]. Among those, CD9, CD82 and Tspan8 have critical roles in cancer metastasis manifested as altered expression levels [13]. In this study, we investigated the changes in the levels of cell adhesion molecules EpCAM, claudins, E-cadherin, tetraspanins, and PKCs at both protein and mRNA level after treating the colon cancer lines with PKC inhibitors (Bisindolylmaleimide I, Gö6976, and Rottlerin). In addition, we sought to determine whether and how the inhibition of PKCs affects cell viability, intracellular ROS signalling and related mechanisms in colon cancer progression.

## Materials and methods

### Cell culture and treatments

Human colon cancer cell lines (Caco-2, DLD-1 and SW620) and normal colon cell line CCD18Co were obtained from ATCC (Manassas, VA, USA). CCD18Co and Caco-2 colon adenocarcinoma cells were maintained in DMEM (Dulbecco’s Modified Eagle Medium), and colon cancer cells (SW620, DLD-1) were maintained in RPMI-1640 medium, supplemented with 10% fetal bovine serum (FBS), 2 mM glutamine, and 1% penicillin/streptomycin. All lines were cultured at 37 °C in a humidified 5% CO_2_ environment. For drug treatments, Bis-I, Gö6976 and Rottlerin were added to the culture media in different concentrations ranging from 2–10 μM and incubated for 24 h. All experiments were performed in triplicate. Passage 4 cells were resuscitated from liquid nitrogen stocks and cultured for a maximum period of 2 months before reinitiating culture from the same passage. ATCC had authenticated the cell lines using morphology, karyotyping, and PCR-based approaches.

### Immunoblotting analysis

After treatment with desired concentrations of Bis-I, Gö6976, and Rottlerin, cells were harvested and washed in PBS. The extracts were prepared by resuspending cell pellets in lysis buffer (50mM Tris-HCI, pH 8.0, 150 mM NaCI, 1% Triton X-100, 5 mM EDTA), briefly sonicated and centrifuged at 15000xg for 15 min at 4°C. The protein concentrations were determined using BCA assay (Thermo). SDS-PAGE and immunoblotting were performed. Nitrocellulose membranes were probed with anti-PKCα, anti-PKCδ, anti-PKCη, anti-PKCγ, anti-PKCζ, anti-GAPDH (sc-208, sc-213, sc-215, sc-211, sc366126, sc-25778, Santa Cruz Biotechnology), anti-PKCβ, anti-PKCϵ, anti-claudin-1, −3, −4, and −7, anti-CD9, anti-CD82, Tspan-8 (Abcam), anti-caspase 3 and 9, anti-LC3A/B, Anti-Atg-5, anti-EpCAM, anti-E-cadherin, anti-vimentin (Cell Signaling). Immunoblotting signals were developed using chemiluminescence. All experiments were performed in triplicate.

### qRT-PCR analysis

Total RNA was isolated from cultured cells using TRI Reagent (Ambion) or Genezol (Geneaid), and the concentration of RNA was determined by Nanodrop (Thermo). 1 μg RNA was used in 20 μl first strand reactions per cDNA synthesis, which was conducted with the High Capacity RNA-to-cDNA Kit. For quantitative RT-PCR, SYBR Green master mix (Applied Biosystems) was used. Fold changes in transcript abundance were calculated by the 2^−ΔΔCt^ method using α-actin as the internal standard. All experiments were performed in triplicate.

### Assessment of cellular viability using MTT assay

The cytotoxic effect was measured using MTT (3-(4,5-dimethylthiazol-2-yl)-2,5-diphenyltetrazolium bromide) as directed by the manufacturer. Briefly, cells were seeded in 96 wells plates at a density of 7500 cells/well. Then, cells were incubated at 5% CO_2_ and 37°C overnight. After treating the cells with PKC inhibitors at different concentrations (2–10 μM) for 24h, MTT was administered into each well for 4 hours at 37 °C, and the absorbance was determined at 540 nm. All experiments were performed in triplicate.

### Cell adhesion assay

For adhesion assay, collagen I coated 96 well-plates were used. Cells were suspended in serum-free culture medium without or with the Bis-I (4 µM) and Rottlerin (2 µM). Then, 100 µL of cell suspension were loaded (n = 4) onto coated wells at 8 × 10^4^ cells/well and incubated at 37 °C in 5% CO2 for 1 h. Ca++/Mg++ containing PBS was used to remove unbound cells. 100 µL of 0.2% crystal violet in 10% ethanol was added to each well, and incubated at room temperature for 5 min. After rinsing each well with PBS, 100 µL of a 50/50 mixture of 0.1 *M* NaHPO4 pH 4.5 and 50% ethanol was added as solubilization buffer. The absorbance was determined at 570 nm as an indication of number of cells attached to the collagen surface. All experiments were performed in triplicate.

### Detection of autophagic punctas by confocal microscopy

Cells were grown on glass coverslips and ﬁxed for 10 min with methanol at −20 °C. After blocking nonspeciﬁc antibody binding with 1% bovine serum albumin (BSA) in PBST (PBS with 0.1% Tween 20), cells were incubated for 1 h with LC3A/B antibody and washed in PBST. Thereafter, they were incubated with secondary antibodies conjugated with Alexa Fluor 594 for 1 h. Cells were washed in PBST and then cell nucleus was stained with DAPI for 5 min at room temperature. At the last step, cells were mounted upside down on the microscope slide in mounting medium to prevent photobleaching. The three-dimensional localization of studied molecules was assessed with confocal microscopy.

### Apoptosis assessment by Annexin V/PI dual staining

In order to evaluate the apoptosis, phosphatidylserine (PS) exposure was analyzed using Annexin V and PI dual staining assay. Annexin V-FITC Apoptosis Detection Kit (Biovision) was used in accordance with the manufacturer’s protocol. After treatment of cells with the indicated PKC inhibitors, the cells at 2.5 × 10^6^ cells/ml were harvested, washed in cold PBS and resuspended in 1X Annexin V Binding Buffer. 5 µl Annexin V-FITC Conjugate and 5 µl Propidium Iodide (PI) Solution was added into each cell suspension. After incubation for 5 min at room temperature in the dark, apoptosis was immediately analyzed by flow cytometry. All experiments were performed in triplicate.

### Measurement of caspase-3 and caspase-9 activities

Cells were seeded on culture plates with PKC inhibitors for 24h and control plates were cultured without inhibitors. After treatment, cells were trypsinized, resuspended in chilled Cell Lysis Buffer, and incubated on ice for 10 minutes. Afterwards, 2X Reaction Buffer (containing 10 mM DTT) and 1 mM DEHD-AFC (caspase-3 activity) and LEHD-AFC (caspase-9 activity) substrate were added to each sample, and then incubated at 37°C for 2 hours. The activity of caspase-3 and caspase-9 was measured by a fluorometer equipped with a 400-nm excitation filter and 505-nm emission filter.

### Detection of intracellular ROS production

25,000 cells per well were seeded in a dark, clear bottom 96-well microplate and cells were allowed to adhere overnight. After removing the media, each well was washed with 1X Buffer. Diluted DCFDA solution was added to stain the cells and cells were incubated for 45 minutes at 37°C in the dark. DCFDA solution was removed and each well was washed with 1X Buffer or 1X PBS. The flavonoids were diluted in 1X Buffer and diluted compounds were added into each well. After 4 hours, fluorescence intensity of each well was measured immediately at Ex/Em = 485/535 nm.

### TBARS assay

5x10^6^ cells were harvested and sonicated in 200 µL of ice-cold PBS. After adding ice-cold 10% TCA to each sample, the samples were incubated for 5 minutes on ice. The samples were centrifuged for 5 min at 14,000 rpm. After mixing TBA Reagent with supernatant, the mixture was vortexed and incubated at 100°C for 60 min. Then the mixture was cooled down to room temperature and loaded to the wells of a black flat-bottom 96-well plate and fluorescence intensity (λex/em = 560 nm/585 nm) was read. MDA standard was used to construct a standard curve.

### Protein carbonyl assay

Per assay, cell lysate containing approximately 0.5–2 mg of protein was used. DNPH was added to each sample, vortexed and incubated for 10 min at room temperature. Into each sample, TCA was added, vortexed, placed on ice for 5 min, and spinned at maximum speed for 2 min. Then, cold acetone was added into each tube and the pellet was washed. The tubes were placed in sonicating bath for 30 seconds. The samples were placed at −20°C for 5 min and then centrifuged for 2 min and acetone was carefully removed. Guanidine solution was added and the pellet was sonicated briefly. The samples were spinned very briefly to pellet any unsolubilized material. The absorbance was measured OD at ~ 375 nm.

### Statistical analysis

Statistical comparisons for gene expression data were conducted in all groups using Microsoft Excel Student’s t-test and *p* values less than 0.05 were regarded as significant. For comparing significant differences between the study groups, one-way ANOVA and Tukey’s multiple comparison test were performed.

## Results

### The expression of cell adhesion molecules (CAM) in colon cancer cell lines

**Figure 1.**
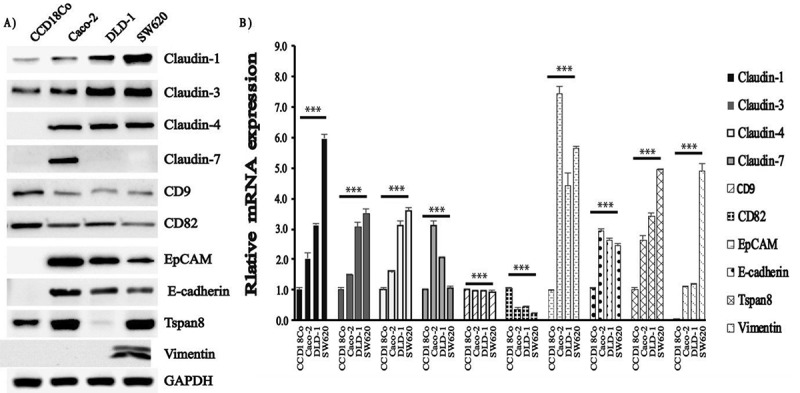
The levels of cell adhesion molecules differed according to the characteristics of the cell lines. (a) Following protein isolation and quantitation, immunoblotting analysis of lysates prepared from CCD18Co, Caco-2, DLD-1, and SW620 cell lines was probed with anti-claudin-1, −3, −4, −7, CD9, CD82, EpCAM, E-cadherin, Tspan8 and GAPDH (loading control). (b) Total RNA was isolated using Trizol Reagent from indicated cell lines, and analysed by qRT-PCR for claudin-1, −3, −4 and −7, CD9, CD82, EpCAM, E-cadherin, and Tspan8 mRNA abundance and α-actin was used as housekeeping gene. Experiments were performed in triplicate. Error bars = SD.; ns = p not significant; * = p < 0.05; ** = p < 0.01; *** = p < 0.001; Students t-test.

To determine the expression of cell adhesion molecules, we performed immunoblotting and qRT-PCR analysis for claudin-1, −3, −4 and −7, EpCAM, E-cadherin, vimentin, Tspan8, CD9 and CD82 in normal human colon cells CCD18Co, colon adenocarcinoma cell lines Caco-2 and DLD-1, and metastatic colon cancer cell line SW620. Caco-2, DLD-1 and SW620 present epithelial morphology while CCD18Co has fibroblast morphology. Immunoblotting results showed that expression of CAM molecules, such as claudin-1, −3 and −4, EpCAM, E-cadherin, and Tspan8 were very low in normal colon cancer cell line CCD18Co compared to Caco-2, DLD-1 and SW620 colon cancer cell lines but no expression was observed in DLD-1 cells for Tspan8. However, expression of another CAM molecule CD82 (KAI1) decreased in colon cancer cells although it was highly expressed in CCD18Co cell line. We also found out that claudin-7 was detected only in Caco-2 cell lines and vimentin was expressed only in SW620. Immunobloting showed the protein level expression of CD9 in the studied colon cell lines albeit at very low levels (Figure 1(a)). The immunofluorescence studies also revealed the membranous localization of the studied molecules which were expressed in the cells (data not shown). However, mRNA expression levels were not totally in line with immunoblotting results for claudin-7, EpCAM, Tspan8 and vimentin (Figure 1(b)).

### Differential expressions of PKC isoforms between colon cancer cell lines

We examined the levels of PKC isoforms in colon cancer compared with colon epithelial cell line by immunoblotting. In addition, mRNA levels of PKC isoforms were next examined using qRT-PCR in colon cancer cells and colon epithelial cell.

**Figure 2.**
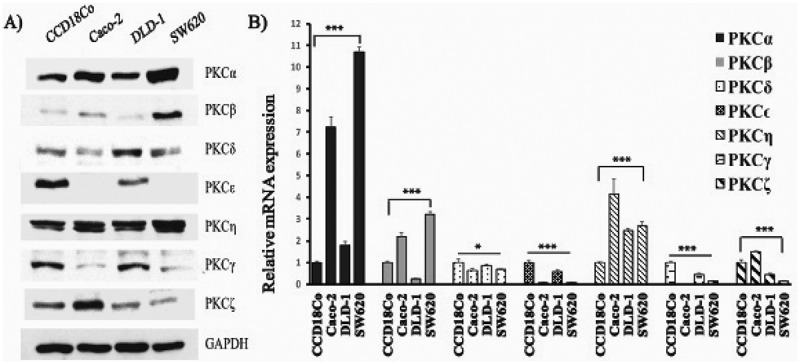
Protein and mRNA expression levels of PKC isoforms in colon cancer cell lines. (a) Immunoblotting analysis of lysates prepared from CCD18Co, Caco-2, DLD-1, and SW620 cell lines probed with anti-PKCα, β, γ, η, ϵ, δ, ξ and GAPDH (loading control). (b) Total RNA was harvested from indicated cell lines, cDNA was synthesized and analyzed by PCR for PKCα, β, γ, η, ϵ, δ, and ξ transcript abundance, and α-actin was used as housekeeping gene. Experiments were performed in triplicate. Error bars = SD.; ns = p not significant; * = p < 0.05; ** = p < 0.01; *** = p < 0.001; Students t-test.

Immunoblotting analysis (Figure 2(a)) revealed that protein levels of PKCα and η upregulated in colon cancer cell lines, Caco-2, DLD-1, and SW620 compared to colon epithelial cell line CCD18Co. We also observed that PKCβ was very low level in the normal colon cell line CCD18Co, primary colon Caco-2, and adenocarcinoma DLD-1 cancer cell lines and but sharply elevated in metastatic colon cancer cell line SW620. On the other hand, we found that expression of PKCγ and ϵ sharply decreased in colon cancer cell lines Caco-2 and SW620 (PKCϵ was undetectable in these cell lines) but both DLD-1 and CCD18Co cell lines expressed almost the same level of these proteins. Moreover, expression of PKCδ increased in both SW620 and DLD-1, but decreased in Caco-2, and PKCζ was expressed at the highest level in Caco-2, conversely very low level in SW620 and DLD-1. Further, we analysed mRNA level of PKC isoforms in these cell lines and qRT-PCR results were consistent with immunoblotting findings except PKCδ and PKCη. We observed that mRNA expression of PKCδ slightly decreased in colon cancer cells (Figure 2(b)). The band intensities of PKCs in the colon cell lines obtained from western blot analysis were shown Figure S2.

### PKC inhibitors enhanced cytotoxicity in colon cancer cells

Earlier studies indicated that Bis-I [15] and Gö6976 [16] are potent and selective PKC inhibitors, especially Bis-I inhibits PKCα, β and γ, and Gö6976 selectively inhibits PKCα and β. On the other hand, Rottlerin is a putative inhibitor of PKCδ. To determine whether PKCs play a role in the survival of colon cancer cells, we cultured CCD18Co, Caco-2, DLD-1, and SW620 cell lines in the absence or presence of Bis-I, Gö6976 or Rottlerin at different concentrations (2–10 μM) for 24 hours.

As presented in Table 1, Bis-I and Rottlerin caused significant cytotoxic effect in CCD18Co, Caco-2, DLD-1, and SW620 cells. Except Gö6976, treatment with the studied PKC inhibitors in the range of 2 and 10 μM strongly induced cytotoxic activities in colon cancer cell lines. After treating the cells with Gö6976 at the same concentration ranges we used for Bis-I and Rottlerin, we detected cellular viability under 50% for cancer cell lines Caco-2, DLD-1, SW620 and even for colon epithelial cell line CCD18Co after 24 h exposure. IC_50_ value for Gö6976 was 10 μM. For both Caco-2 and DLD-1 cell lines, Bis-I and Rottlerin had cytotoxic activity at concentrations ranged between 2–4 μM (Table 1). However, after 48 and 72h, the PKC inhibitors did not show cytotoxic effect on the studied cells (data not shown). Therefore, PKC inhibitors were used for 24 h in the subsequent experiments.

**Table 1.** In vitro cytotoxicity of Bis-I, Gö6976 or Rottlerin. IC_50_ values of Bis-I, Gö6976, and Rottlerin in CCD18Co, Caco-2, DLD-1, and SW620 cell lines were determined. Results are presented as mean ± standard deviation of 3 independent experiments.

	IC_50_ values (µM)			
	CCD18Co	Caco-2	DLD-1	SW620
Bis-I	4 ± 0.5	4 ± 0.2	2.5 ± 0.1	4 ± 0.6
Gö6976	10 ± 1.5	10 ± 1.1	10 ± 1.4	10 ± 1.2
Rottlerin	2 ± 0.4	4 ± 0.7	2 ± 0.2	2 ± 0.4

### Treatment with PKC inhibitors affected the expression level of CAM in colon cancer

Following the findings that levels of PKCs and CAM altered between normal and tumor cells, we sought to understand the relationship between the expression of PKCs and the expression of CAM. Based on the IC_50_ values from the cytotoxicity analysis (Table 1), we determined the optimum the concentrations of Bis-I (4 μM), Gö6976 (10 μM) and Rottlerin (2 μM) for further studies. We treated both colon cancer cell lines Caco-2, DLD-1 and SW620, and colon epithelial cell line CCD18Co with the indicated concentrations of PKC inhibitors. After 24 hours, the cells were harvested, qRT-PCR and immunoblotting analysis were conducted.

**Figure 3.**
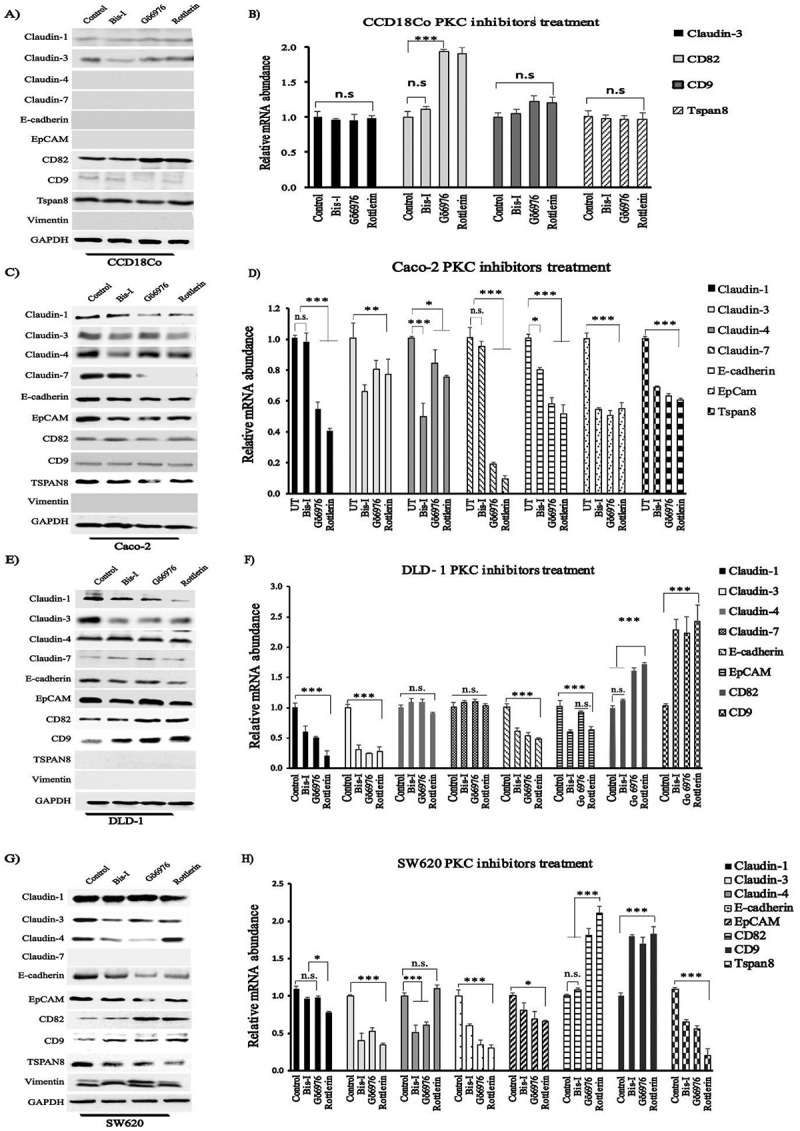
PKC inhibitor treatment selectively changed expression of cell adhesion molecules in colon cancer cells. CCD18Co, Caco-2, DLD-1 and SW620 cells were treated with Bis-I, Gö6976 and Rottlerin. Following PKC inhibitors treatment for 24 h, total RNA was harvested, cDNA was synthesized and analyzed by PCR for indicated cell adhesion molecules. Cells were analyzed for indicated cell adhesion molecules protein abundance by immunoblotting. Western blot and qRT-PCR data for CCD18Co (a), Caco-2 (b), DLD-1 (c) and SW620 (d) cells. Experiments were performed in triplicate. Error bars = SD.; ns = p not significant; * = p < 0.05; ** = p < 0.01; *** = p < 0.001; Students t-test.

Immunoblotting indicated that claudin-1 and −3, CD9 and Tspan8 did not alter either in the absence or presence of PKC inhibitors while the expression of CD82 increased after treatment of CCD18Co cells with Gö6976 and Rottlerin (Figure 3(a)). Protein abundance of other studied CAM genes in CCD18Co cells was undetectable in the presence of PKC inhibitors. When qRT-PCR was used to measure relative transcript abundance, we detected an increase in the expression of CD82 in CCD18Co cells, but no significant changes in the expressions of claudin-1, CD9 and Tspan8 (Figure 3(b)). In Figure 3(b), Claudin-1, CD82, CD9 and Tspan8 mRNA abundances were shown in CCD18Co cell line but the transcript abundances of other studied CAM genes were not shown due to either undetectable mRNA level or no significant change. Following PKC inhibitor treatment, we observed significant decrease in protein (Figure 3(c,e,g)) and mRNA (Figure 3(d,f,h)) levels of claudin-1, EpCAM, E-cadherin in Caco-2, DLD-1 and SW620 cells, except for claudin-1 in SW620 cells. Treatment with PKC inhibitors resulted in significant decrease in the expression of claudin-3 and −4 in Caco-2, DLD-1 and SW620 (except Rottlerin treatment in SW620). We also observed that expression of CD9 and CD82 increased both at mRNA and protein level following the treatment with PKC inhibitors in DLD-1 and SW620 cell lines (Figure 3(e-h)). On the other hand, although CD9 and CD82 were detectable at the protein level (Figure 3(c)), no change was observed for the expression of these genes at mRNA level in Caco-2 cells after treatment with PKC inhibitors. Protein and mRNA levels of Tspan8 were at undetectable levels in DLD-1 cells (Figure 3(e)), but the protein and mRNA abundance decreased following the treatment with PKC inhibitors in Caco-2 and SW620 cells (Figure 3(c,d,h,g)). Claudin-7 was only detectable in Caco-2 cells, also in DLD-1 though not significant, and markedly decreased after Gö6976 and Rottlerin treatment in Caco-2 cells (Figure 3(c,d)). SW620 was the only cell line expressing vimentin, but we did not observe any change in the transcript level of vimentin following the treatment with PKC inhibitors (Figure 3(g)). The band intensities (western blot Figure 3) of CAMs in the PKC inhibitor-treated colon cell lines were shown in Figure S3.

### Effect of PKC inhibitors on cell adhesion to collagen

**Figure 4.**
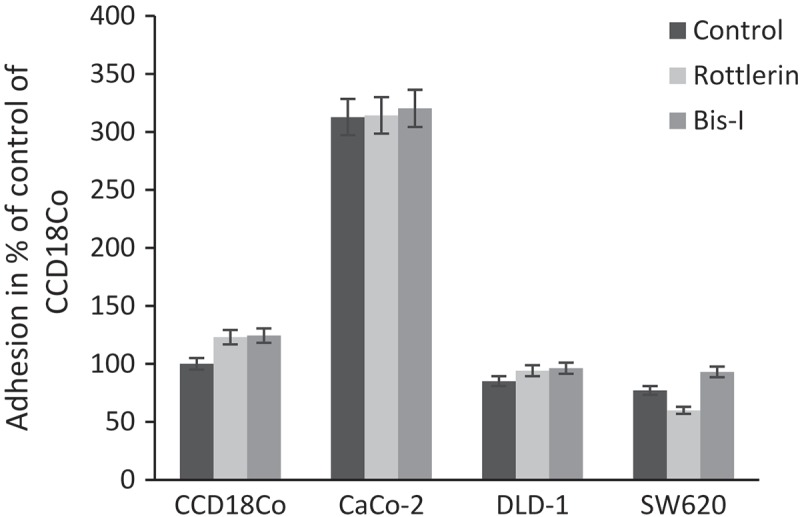
Cell adhesion to collagen after PKC inhibition. Experiments were performed in triplicate. Error bars = SD.

The PKCs also mediate cell adhesion; therefore, to investigate the adhesion of the studied colon cells to ECM proteins, cell adhesion assay on collagen coated plate was performed. The colon cell lines were cultured in serum-free growth medium containing or not containing PKC inhibitors Rottlerin (2 µM) and Bis-I (4 µM) for 2 h. A percentage adhesion value was calculated according to the absorbance of untreated (control) normal colon cells, CCD18Co. As shown in Figure 4, incubation with or without Rottlerin and Bis-I resulted in activation of cell adhesion in Caco-2 cells, and inhibition of cell adhesion in DLD-1 and SW620 cells compared to CCD18Co. There were 23%, 40% and 7% adhesion inhibition for untreated, Rottlerin and Bis-I treated metastatic colon cancer cell, SW620 compared to untreated normal colon cell, CCD18Co. However, when we compare the cell adhesion among themself, only SW620 with Rottlerin treatment showed reduction in the number of adherent cells on the collagen and there was minor alteration in Caco-2 and DLD-1 cells without any significance.

### Autophagy was activated by PKC inhibition

The cross-talk between autophagy and cell adhesion molecules as well as the potential involvement of PKCs in this crosstalk have not yet been described. Thus, we next examined the proteins which have important roles in apoptosis and autophagy of the cell lines used in this study either in the absence or presence of PKC inhibitors.

**Figure 5.**
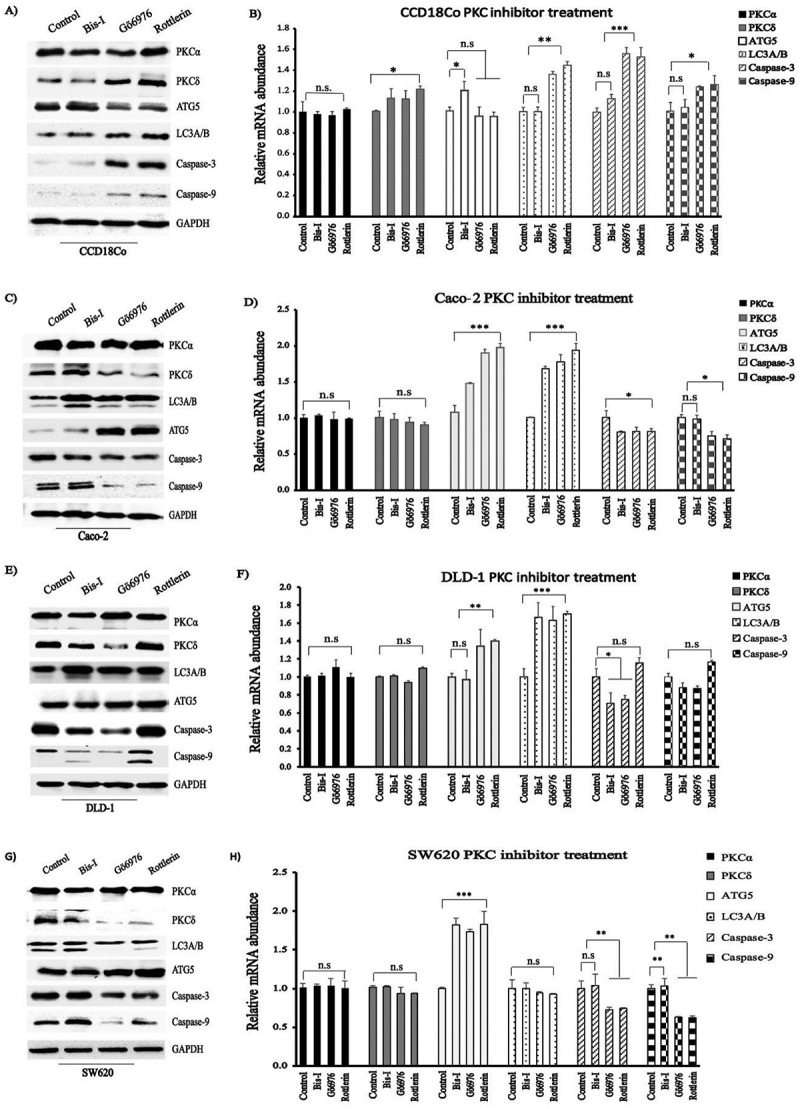
Apoptotic and autophagic genes were altered via PKC inhibitor treatment. (a, c, e, g). the cells-treated with or without PKC inhibitor were immunoblotted for PKCα and δ, ATG5, LC3A/B, caspase-3 and −9, and GAPDH. (b, d, f, g). qRT-PCR analysis of relative mRNA abundance of indicated genes in control and PKC inhibitor-treated cells. Experiments were performed in triplicate. Error bars = SD.; *p < 0.05; **p < 0.01; ***p < 0.001; Student t-test.

We identified a significant increase in LC3A/B protein levels, which is an autophagy marker, after treatment with PKC inhibitors in CCD18Co (Figure 5(a)), Caco-2 (Figure 5(c)) and DLD-1 (Figure 5(e)) cells and no change in SW620 cells (Figure 5(g)). Performing qRT-PCR analysis revealed gene expression levels in line with the protein levels we measured (Figure 5(b,d,f,h)). Similarly, we observed measurable increase in ATG5 transcript abundance following the treatment with PKC inhibitors in Caco-2, DLD-1 (except Bis-I) and SW620, and no significant change in mRNA levels in CCD18Co cell line after Gö6976 and Rottlerin treatment, but significant change for Bis-I treatment (p < 0.05). We detected visible decreased protein level of Atg5 after Gö6976 and Rottlerin treatment in CCD18Co cell line (Figure 5(a)). The band intensities (western blot Figure 5) of autophagic genes in the PKC inhibitor-treated colon cell lines were shown Figure S4.

**Figure 6.**
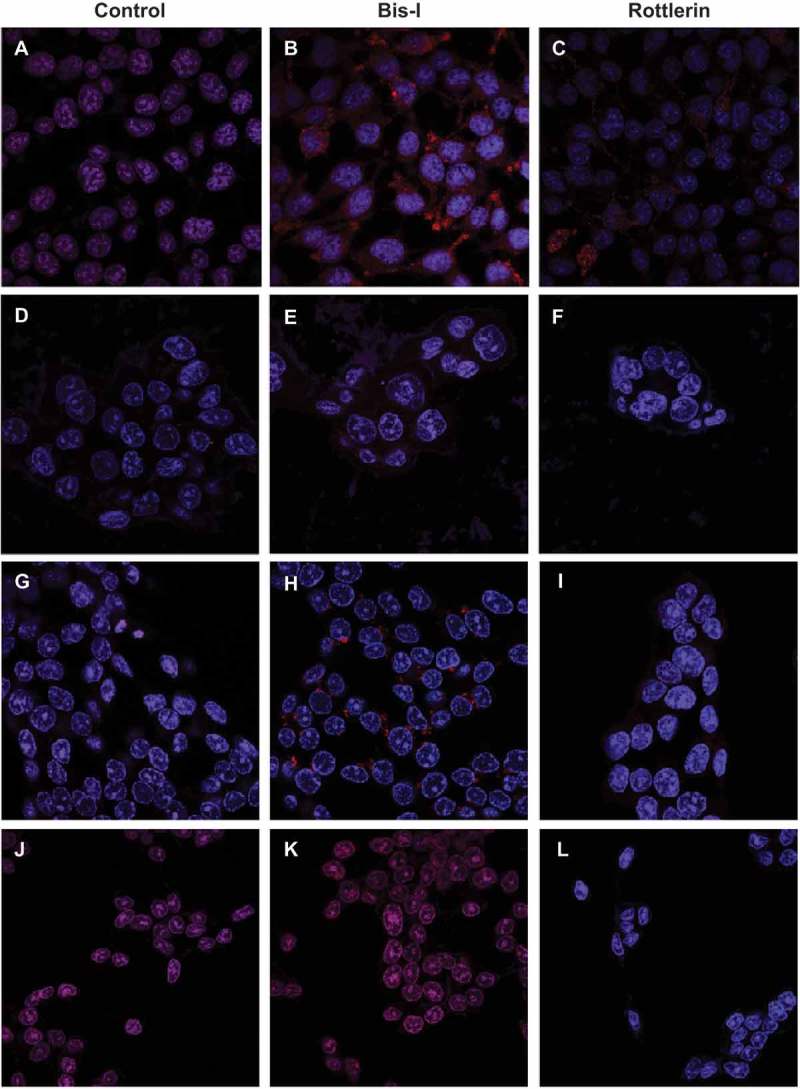
The effects of PKC inhibitors on the autophagosomes formation. (a-c) represent CCD18Co cells, (d-f) represent Caco-2 cells, (g-i) represent DLD-1 cells and (j-l) represent SW620 cells. First column shows untreated (control), second column shows Bis-I treated and third column shows Rottlerin treated cells.

Induction of autophagy after treatment with Rottlerin and Bis-I was monitored by the conversion of LC3-I to LC3-II, clustering of LC3 into dot-like cytoplasmic structures. PKC inhibitors dramatically induced LC3 processing and autophagosome formation as shown in Figure 6. We found that LC3 staining showed a small amount of autophagosomes at the basal condition, especially in normal colon cells, CCD18Co, although autophagosome formation was not seen in the absence of PKC inhibitors. However, autophagy was greatly induced by Bis-I and Rottlerin treatment in CCD18Co cells. In addition, treatment with 4 μM of Bis-I resulted in a significant increase in the formation of autophagosome especially in CCD18Co and DLD-1 cells when compared to Rottlerin treatment and control.

### PKCs inhibited apoptosis

The underlying mechanisms how PKC signalling alters apoptosis remain still controversial. We assessed the caspase-3 and −9 at mRNA and protein levels in the studied cell lines (Figure 5). In CCD18Co cells, mRNA and protein levels of both Caspase-3 and −9 were upregulated after Gö6976 and Rottlerin treatment but the expression of these genes was stable after treatment with Bis-I. On the other hand, both Caspase-3 and 9 were either stable or downregulated in Caco-2 and SW620 cell lines following the treatment with PKC inhibitors. In DLD-1 cells, we observed a decrease in the expression of these genes following Bis-I and Gö6976 treatment and an increase following the treatment with Rottlerin. All together, these findings revealed that the inhibition of PKCs in colon cancer cells caused cell death via inducing the expression of apoptosis and autophagy genes. The band intensities (western blot Figure 5) of Caspase-3 and −9 in the PKC inhibitor-treated colon cell lines were shown in Figure S4.

**Figure 7.**
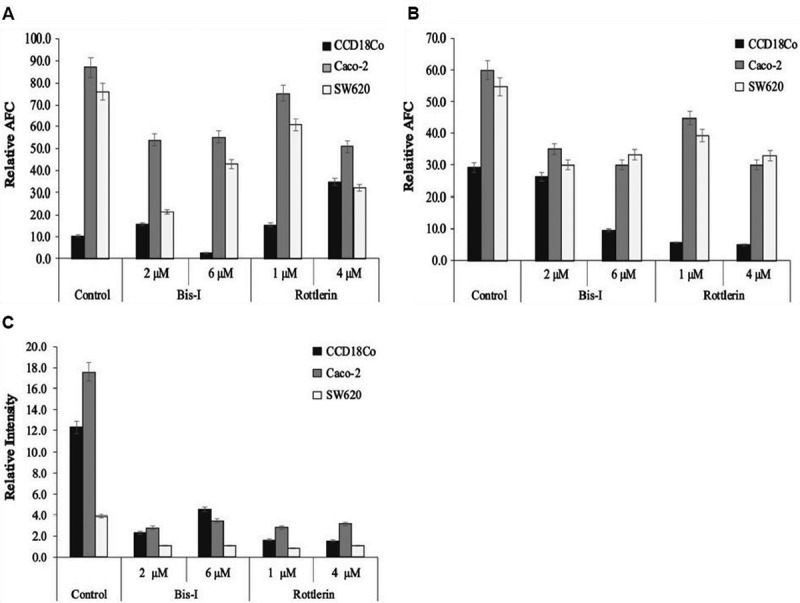
Caspase-3 and −9 activities, and also ROS production modulated by PKCs. After treatment of PKC inhibitors, caspase-3 (a) and −9 (b) activities of CCD18Co, Caco-2 and SW620 cells were measured by using specific fluorophore products after cleavage with fluorometer. (c) Caco-2, DLD-1 and SW620 cells were stained with DCDFA for 30 minutes and treated with Bis-I and Rottlerin. After incubation with Bis-I (2 and 6 µM) and Rottlerin (1 and 4 µM) for 4 h, the fluorescence was measured in a microplate reader. Graphs represent the intensity of DCF in all cells. Experiments were performed in triplicate. Significant differences were calculated using one-way ANOVA followed by Tukey’s multiple comparison test. n = 5, *p < 0.05, **p < 0.01 vs. the control cells.

**Figure 8.**
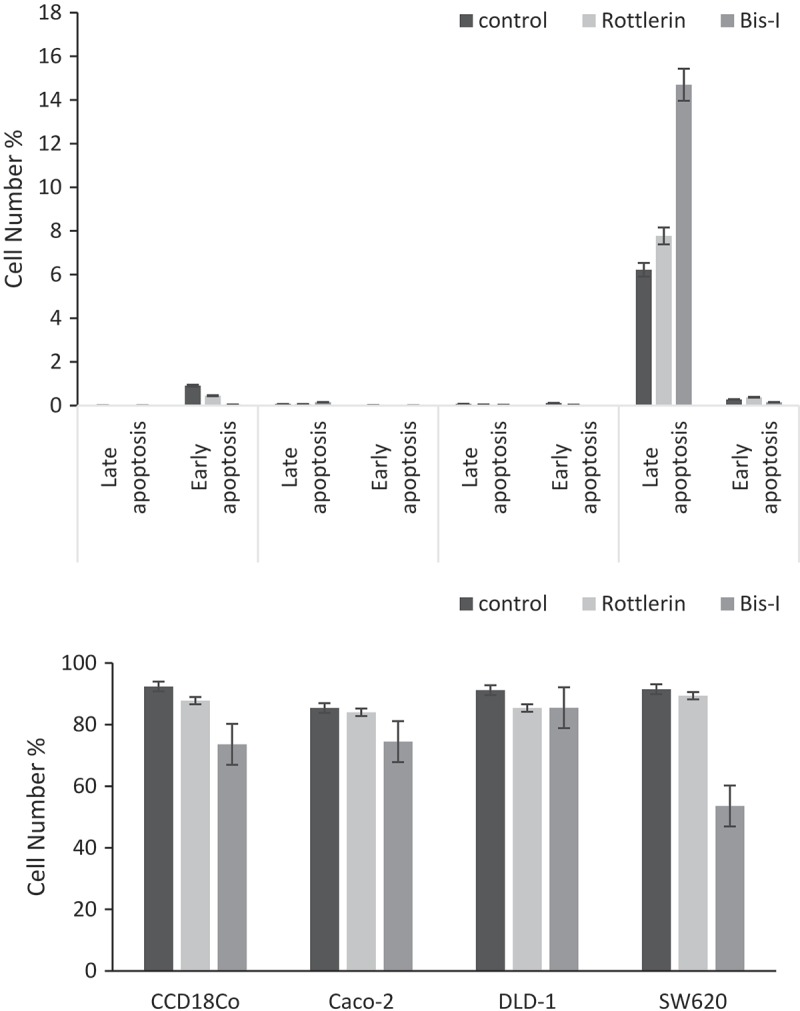
The cell numbers of early, late apoptotic and live cells in CCD18Co, Caco-2, DLD-1 and SW620 cells after Rottlerin and Bis-I treatment. Experiments were performed in triplicate. Error bars = SD.

In parallel to immunoblotting and qRT-PCR analysis, assays measuring the activities of caspase-3 and −9 were performed. Activity of caspases was inhibited and cell viability decreased after the treating the cells with Bis-I or Rottlerin whereas we observed no significant cytotoxic activity for Gö6976 in both colon cancer and normal colon epithelial cell lines. Therefore, Gö6976 was not further used in the subsequent experiments. Caspase-3 and −9 have active roles in all steps of apoptosis. Addition of Bis-I and Rottlerin reduced the activity of caspase-3 in Caco-2 cells compared to control cells (Figure 7(a,b)). In SW620 cells, caspase-3 activities decreased 20 and 58% after 1 µM and 4 µM Rottlerin treatment, respectively while there was a negative correlation between the activity of caspase-3 and the concentration of Bis-I. In contrast, Bis-I and Rottlerin increased caspase-3 activity in CCD18Co cells except in 6 µM of Bis-I-treated cells. After treatment of CCD18Co, Caco-2 and SW620 cell lines with Bis-I and Rottlerin, caspase-9 activities decreased in all studied cell lines compared to control cells. In addition, as shown in Figure 8, the number of dead SW620 cells increased in the presence of Bis-I whereas it decreased or didn’t change in Rottlerin- or Bis-I treated CCD18Co, Caco-2 and DLD-1 cells.

### ROS and PKCs crosstalked in colon cancer progression

We examined the relationship between PKC-apoptosis and the level of reactive oxygen species (ROS). Therefore, we investigated whether PKC inhibition may stimulate/inhibit cellular ROS and thus ROS generation may in turn activate/inactivate PKC and provide signal for carcinogenesis/apoptosis.

DCF fluorescence was recorded by fluorescence microplate reader to measure the intracellular production of ROS in control and inhibitor-treated cells. As shown in Figure 7(c), treatment of the cells with PKC inhibitor, Bis-I reduced the intracellular ROS levels by 81.4, 84.2 and 73% in CCD18Co, Caco-2 and SW620 cells, respectively, even at the low concentration (2 µM) of Bis-I, compared to the control cells. Similarly, the mean fluorescence in the cells significantly decreased after Rottlerin treatment. The reduction of ROS production upon PKC inhibition can be explained by the suppression of caspase-3 and −9 activities, which were activated by ROS-induced apoptosis.

### PKC inhibition decreased the levels of ROS-damaged biomolecules

The eukaryotic cells are evolutionarily evolved to modulate the oxidant levels [17]. However, in the case of persistent exposure to the oxidants and other toxic agents, excessive ROS are produced that can cause oxidative damage to biomacromolecules such as peroxidation of membrane lipids, oxidation of amino acid side chains (especially cysteine), formation of protein-protein cross-links, oxidation of polypeptide backbones resulting in protein fragmentation, DNA damage, and DNA strand breaks [18,19]. To further analyse the interrelation among ROS-PKC-apoptosis, we determined the alterations in the levels of the ROS-damaged biomolecules in PKC inhibited conditions.

**Figure 9.**
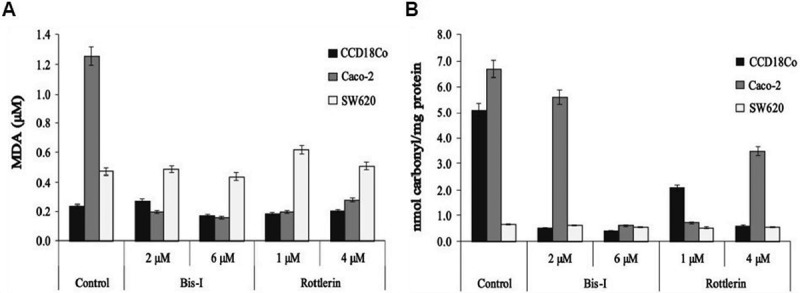
MDA and protein carbonyl levels decreased with PKC inhibition. The cells were treated with the indicated concentrations of PKC inhibitors for 24 hours and then MDA (a) and protein carbonyl (b) levels were measured by TBARS Assay Kit and Protein Carbonyl Assay Kit, used per manufacturer’s instructions, respectively. Experiments were performed in triplicate. Error bars = SD.

The damaging effect of intracellular ROS in CCD18Co, Caco-2 and SW620 cells decreased following the treatment with PKC inhibitors as revealed by the reduction in the levels of lipid peroxidation and protein oxidation products, MDA and protein carbonyl (Figure 9(a,b)). These results underscore the direct crosstalk among PKC signalling, ROS production and oxidative stress-damaged products in the maintenance of cellular homeostasis.

## Discussion

The processes involved in the maintenance, promotion or disruption of cell adhesion is particularly important in colon cancer progression and metastasis. We have assessed the differences CAMs and PKCs both at protein and mRNA expression levels between colon cancer cell lines and normal colon epithelial cells. Our results demonstrated that claudin-1, −3, −4 and −7, and EpCAM were the important biomarkers in the process of colon carcinogenesis. Similarly, others showed that elevated expression of EpCAM [20] and altered expression of claudins, especially increases in the expression of claudin-1, −3, −4 and downregulation of claudin-7, are closely related to the metastasis process in colon cancer cells [21]. Our results showing the elevated expression of claudin-1, −3 and −4, EpCAM, E-cadherin in colon cancer were consistent with earlier studies [11,22,23] . Previously it was shown that decreased expressions of CD9 [24] and CD82 were vital for colon cancer metastasis, and particularly reduced expression of CD82 was related with metastatic colon [25] and gastric cancer [26]. Here, we have also identified expression changes in seven PKC isoforms in both normal colon cells and colon cancer cells. Our findings uncovered increased expressions of PKCα and η in both adenocarcinoma cell lines (Caco-2 and DLD-1) and metastatic cell line (SW620) compared to normal colon cell line (CCD18Co). On the other hand, we observed that protein and mRNA levels for PKCγ and ε decreased in colon cancer cell lines compared to normal colon cells. Moreover, we found that the expression level of PKCδ, β and ζ presented inconsistent alteration between normal colon and cancer cell lines. Goldstein et al. reported that there was no change in mRNA abundance of PKCα, δ, and η between the normal and cancer tissues [27]. Using immunoblotting, another study identified decreased levels of PKCβ and ϵ, increased level of PKCδ, and stable levels of PKCα and ζ in colon cancer tissues [28]. Pysz et al. showed that both PKCα and PKCδ were downregulated in colon cancer cell lines [29]. Also, there were studies showing the upregulation of PKCδ without any difference in the levels of PKCα in colorectal cancer patients. In addition, another study on primary colon tumors reported decreased total PKC activity and increased expression of PKCδ, and no difference at the level of PKCα between normal tissues and colon tumors. Others have earlier showed that there is no significant difference at the levels of PKCα and δ mRNA between normal colon mucosa and human adenocarcinoma [27,28]. Leotlela et al. observed the changes in PKC activity via PMA treatment increased claudin-1 levels in melanoma cells [30]. Another study showed that the treatment of neuroblastoma cell lines with PKC inhibitors increased cell-cell adhesion [31]. Such findings support the conclusion that PKCs may play important roles in the expression of CAMs. Despite the vast amount of information about modulations of PKC function in cellular models, the relevance of individual PKC isozymes in the progression of human cancer is still a matter of controversy. PKC signaling controls the several signaling pathways in the cell [32–35]. Although PKC inhibitors used in this study affect several pathways in different cellular process, they are able to specifically inhibit their targeted PKC isoform activity. Therefore, PKC inhibitor drugs may impact specific gene expression programs or pathways [36–38]. We investigated how PKC signaling modulates CAM dynamics on the membrane, and cell death such as apoptosis and autophagy inside the cell. Immunoblotting and qRT-PCR data revealed that the levels of cell death markers and CAMs were changed following the treatment with PKC inhibitors, especially in the case of Rottlerin, in both colon epithelial and cancer cell lines. These findings emphasize that the activity of PKCs is vital for cell survival and mechanisms involved in colon carcinogenesis.

Endothelial adhesion of blood vessels of tumor cells during extravasation is one of the most important preconditions for metastasis formation. It is important that cell adhesion to the extracellular matrix is inhibited because the extravasation and intravasation capabilities of the cancer cells are largely controlled by basement membrane and extracellular matrix attachment. We found that only Rottlerin reduced the cell adhesion in metastatic colon cell line, SW620 compared to the normal and primary colon cell lines. This finding suggests that Rottlerin may have the potential to inhibit adhesion of tumour cell following invasion and metastasis. In cultured colon cancer cells, we showed that expression of caspase-3 and −9 significantly decreased after the treatment with PKC inhibitors except Rottlerin treatment in DLD-1 cells and Bis-I treatment compared to normal colon cells CCD18Co, but we observed significant increases in the expression levels of autophagy genes Atg5 and LC3A/B in colon cancer cells except for LC3A/B in SW620. Immunocytochemistry analysis using an antibody against LC3A/B was also used to monitor the autophagosomes formation in colon cells. The results demostrated that Bis-I and Rottlerin promoted autophagic flux, suggesting that the studied PKC inhibitors may regulate autophagy response. Treatment with Rottlerin resulted in decreased expressions of EpCAM, E-cadherin, and claudin-3 both at mRNA and protein levels in all colon cancer cells. Also, these inhibitors suppressed the activities of caspase-3 and −9 in the colon cancer cells studied. These results suggest that PKC inhibitors, particularly Rottlerin, induces autophagy and suppresses apoptosis in colon cancer cells. Moreover, these drugs either diminish or induce expression of CAM, like EpCAM or CD82, which were altered through cancer initiation, progression and metastasis. The decreases in the protein and mRNA levels of caspase-3 and −9 as well as their activities were observed in the Rottlerin-treated Caco-2 and SW620 cells. When compared to immunoblotting and qRT-PCR analysis, the results suggested that PKC inhibition may repress apoptosis and induce autophagic genes at the protein and mRNA levels. Besides, the activity of caspases also decreased. The results we obtained through immunoblotting and qRT-PCR analysis were highly concordant for apoptotic and autophagic genes. In line with our findings, Satoh et al. showed that PKC inhibition by PKC inhibitor IYIAP reduced caspase-3 and −9 activities [39]. When PKC was downregulated by prolonged exposure to phorbol 12,13-dibutyrate or inhibited by staurosporine, calphostin C, or H-7 in VSMC, caspase-3 activity decreased [40]. However, when compared to the all western blot and mRNA expression results it observed differences in some genes. The some conflicting results may be from post-transcriptional mechanisms and in vivo half lives of proteins. Whether alterations in PKC activity enhance or suppress apoptosis appear to depend on the initiating signal as well as the specific cell type. Our results directed us to investigate whether ROS-triggered signalling can be altered in a PKC-dependent manner. We showed that PKC inhibition decreased ROS production and prevented the protein and lipid damage, finally led to apoptosis suppression. The results of this study were consistent with another study showing reduced ROS production in the presence of the PKC inhibitors IYIAP, staurosporine, and chelerythrine and the subsequent suppression of apoptosis associated with the reduced activation of caspases-3 and −9 [39]. Hu et al. observed that the ROS generation can be prevented dramatically by bisindolylmaleimides in HepG2 cells [41]. ROS-triggered signaling can be generated in a PKC-dependent manner and may crosstalk with PKC-mediated pathway [42–44]. Similarly, in thyroid tumors, with high level of oxidative stress, lipid peroxidation product HNE content was significantly higher than the level in matched normal tissue [45]. In other tumour types such as the breast cancers at different degrees of malignancy, HNE immunostaining was strongest in invasive breast carcinomas [46].

All together, these results showed the causal relationship among the expression of PKC isozymes, cell adhesion molecules, cell death-related proteins, and ROS levels through the initiation and progression of colon cancer.

## Data Availability

The datasets used and/or analysed during the current study are available from the corresponding author upon reasonable request.
