# Supplementary material for: Protein kinase C Inhibitors selectively modulate dynamics of cell adhesion molecules and cell death in human colon cancer cells
Source: Cell Adh Migr. 2018 Oct 11;13(1):83–97. doi: 10.1080/19336918.2018.1530933 (PMC6527378; doi:10.1080/19336918.2018.1530933)
Supplement: Supplemental Material [file kcam-13-01-1530933-s001.docx]

**Supporting information**

**Fig. S1.** The protein band intensity of cell adhesion molecules differed according to the characteristics of the cell lines compared to untreated CCD18Co cells. Experiments were performed in triplicate. Error bars=SD.

**Fig. S2.** The protein band intensity of PKC isoforms in colon cancer cell lines compared to untreated CCD18Co cells. Experiments were performed in triplicate. Error bars=SD.

**Fig. S3.** The protein band intensity of cell adhesion molecules in colon cancer cells when treated with Bis-I, Gö6976 and Rottlerin compared to untreated CCD18Co cells. Experiments were performed in triplicate. Error bars=SD.

**Fig. S4.** The protein band intensity of apoptotic and autophagic genes altered via PKC inhibitor treatment compared to untreated CCD18Co cells. Experiments were performed in triplicate. Error bars=SD.
